# Supplementary material for: Quantitative investigation reveals distinct phases in Drosophila sleep
Source: Commun Biol. 2021 Mar 19;4:364. doi: 10.1038/s42003-021-01883-y (PMC7979771; doi:10.1038/s42003-021-01883-y)
Supplement: Supplementary file 2 — Supplementary Information [file 42003_2021_1883_MOESM2_ESM.pdf]

Supplementary Information

Quantitative Investigation Reveals Distinct Phases in Drosophila Sleep

Xiaochan Xu, Wei Yang, Binghui Tian, Xiuwen Sui, Weilai Chi, Yi Rao, Chao Tang*

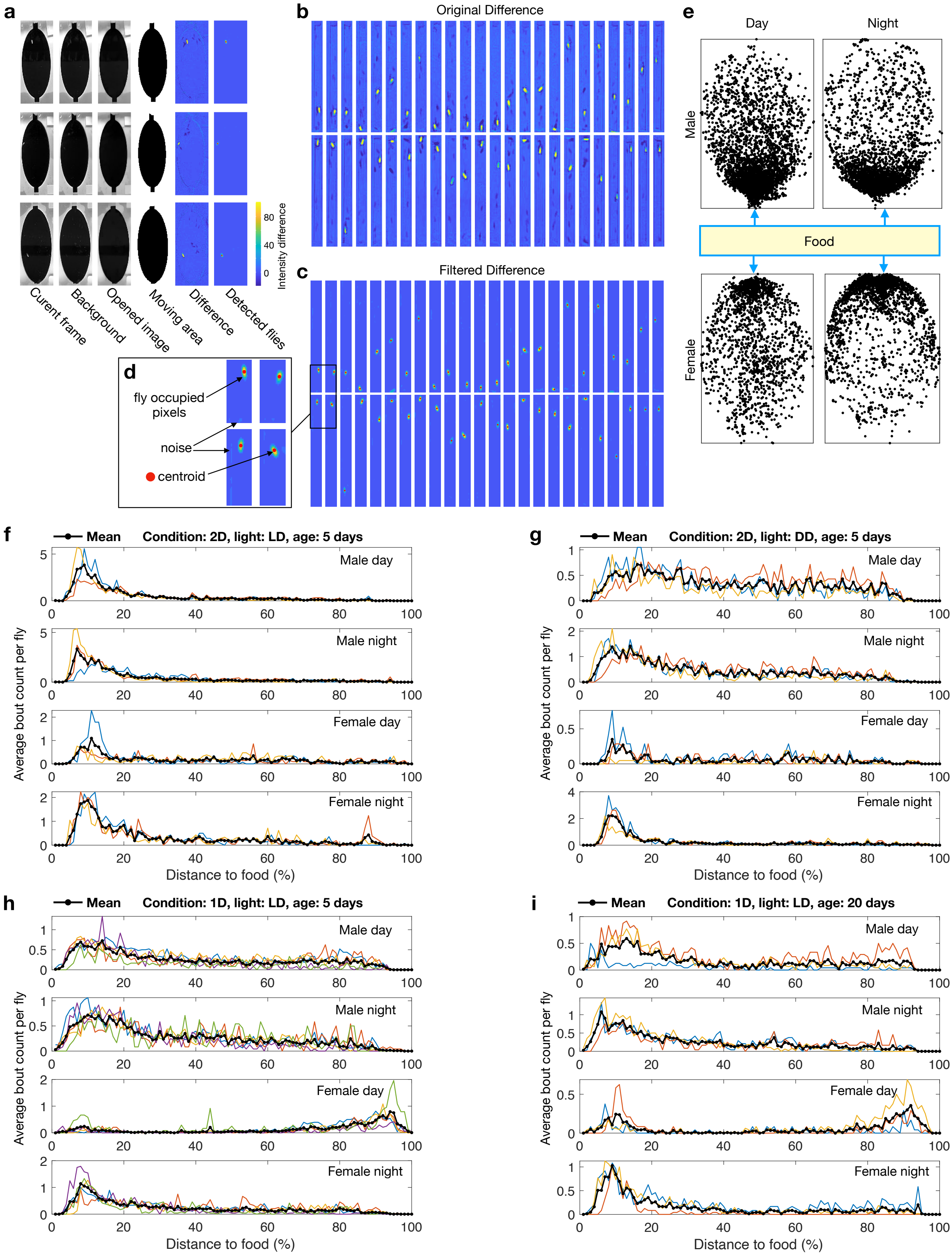

Supplementary Figure 1. High resolution video platform for tracing flies' locomotion activities.
a From left to right: one original frame of a fly, background frame calculated with the mean of 12 frames sampled from 12 hours, opened image after the morphological opening of the background, moving area (black) extracted through binarizing the opened image, difference between the original frame and the background, and flies' current locations within the 2D observing channels. The color key indicates the intensity difference at each pixel. **b** Difference picture obtained by subtracting the background picture from the original picture within the 1D observing channels. **c–d** Filtered difference picture and zoom in for flies occupied pixels. **e** Location preference of fly when sleeping in 2D observing channels in 3 days. Rest bouts of more than 5 min are shown, each black dot represents one sleeping bout. **f–i** Statistics of location preference of fly when sleeping. Colored curves on each plot show the mean count per fly when resting more than 5 min in each experiment repeat in 3 days. The black curves represent the mean value of all the flies. The distance to food is normalized with the length of the observing channels and the food is placed at 0. All of the four different conditions used in the main text are shown.

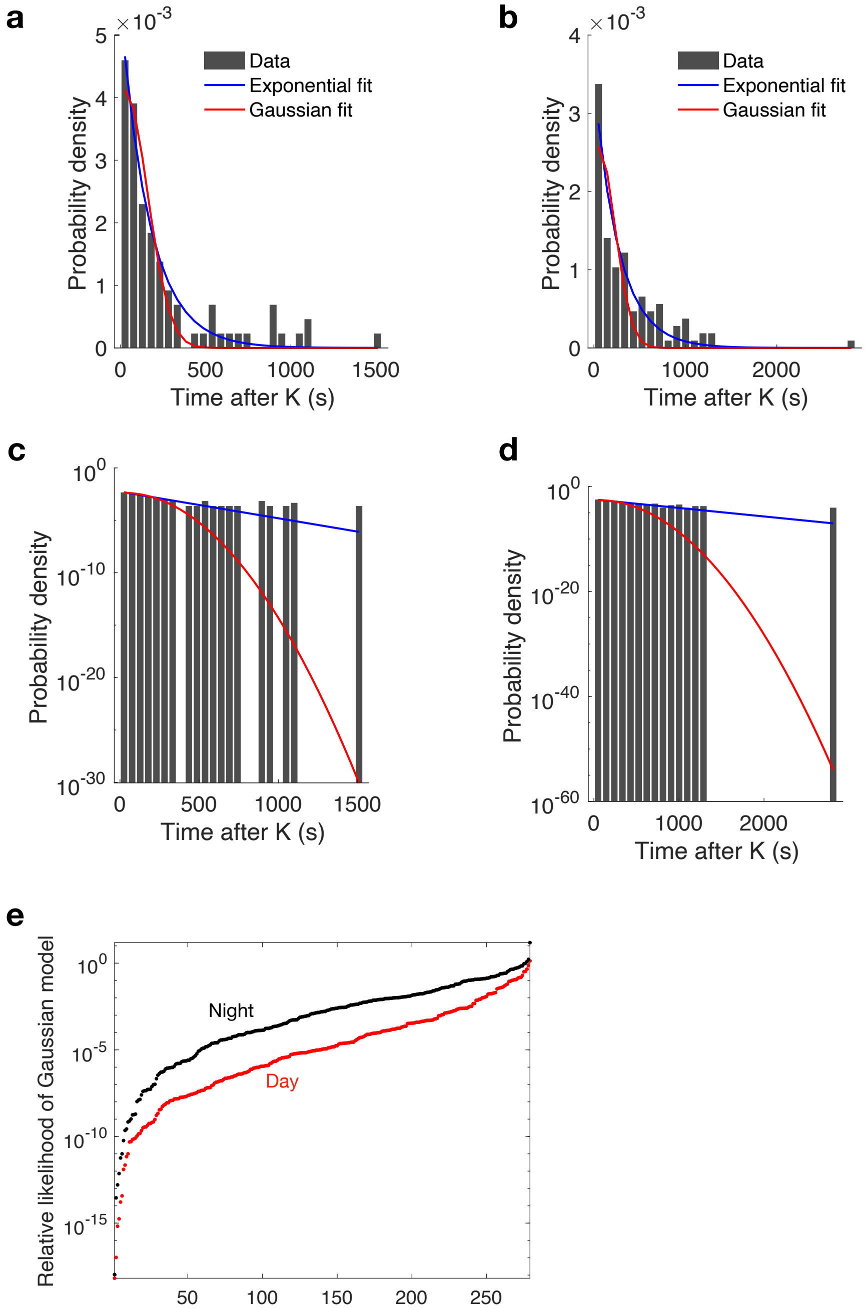

Supplementary Figure 2. Quantitative sleep pattern definition based on the probability distribution of rest-bout duration.
a–**d** Comparison of the exponential fit and gaussian fit for the distribution of the long rest time. Day samples and night samples longer than the sleep latency K(s) from one fly are shown respectively. **a**–**b** used double logarithmic axes, and **c**–**d** used only logarithmic y-axis. **a** and **c** show the day samples. **b** and **d** show the night samples. **e** Relative likelihood of gaussian model calculated with AIC. Relative likelihood = $e^{(AIC_{exp}-AIC_{gauss})/2}$.

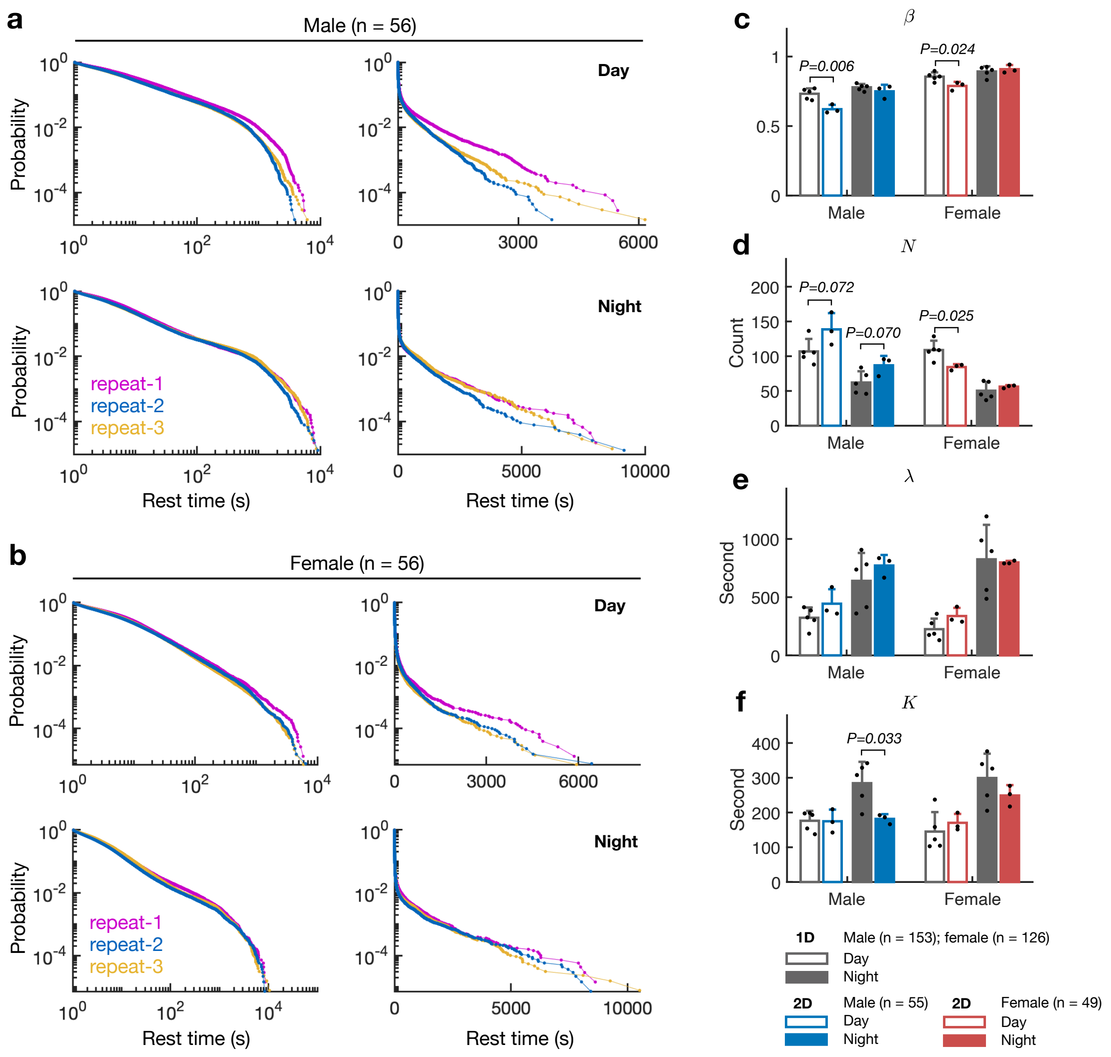

Supplementary Figure 3. Comparison of sleep parameters between the light and dark phases.
a–b Quantitative sleep pattern in 2D observing channels shown with all the male flies' data and all the female flies' data respectively. The probability functions of the three repeats are shown, and n indicates the number of flies used. The first rows are samples in the light phase, and the second rows are samples in the dark phase. The left panels use double logarithmic axes, and the right panels use only logarithmic y-axis. **c–f** Parameter comparison of the quantitative sleep pattern between the 1D and 2D observing channels. The hollow and filled columns represent the mean of the parameter in the light and dark phases, respectively; Black: flies in 1D channels; blue, male in 2D channels; red, female in 2D channels.

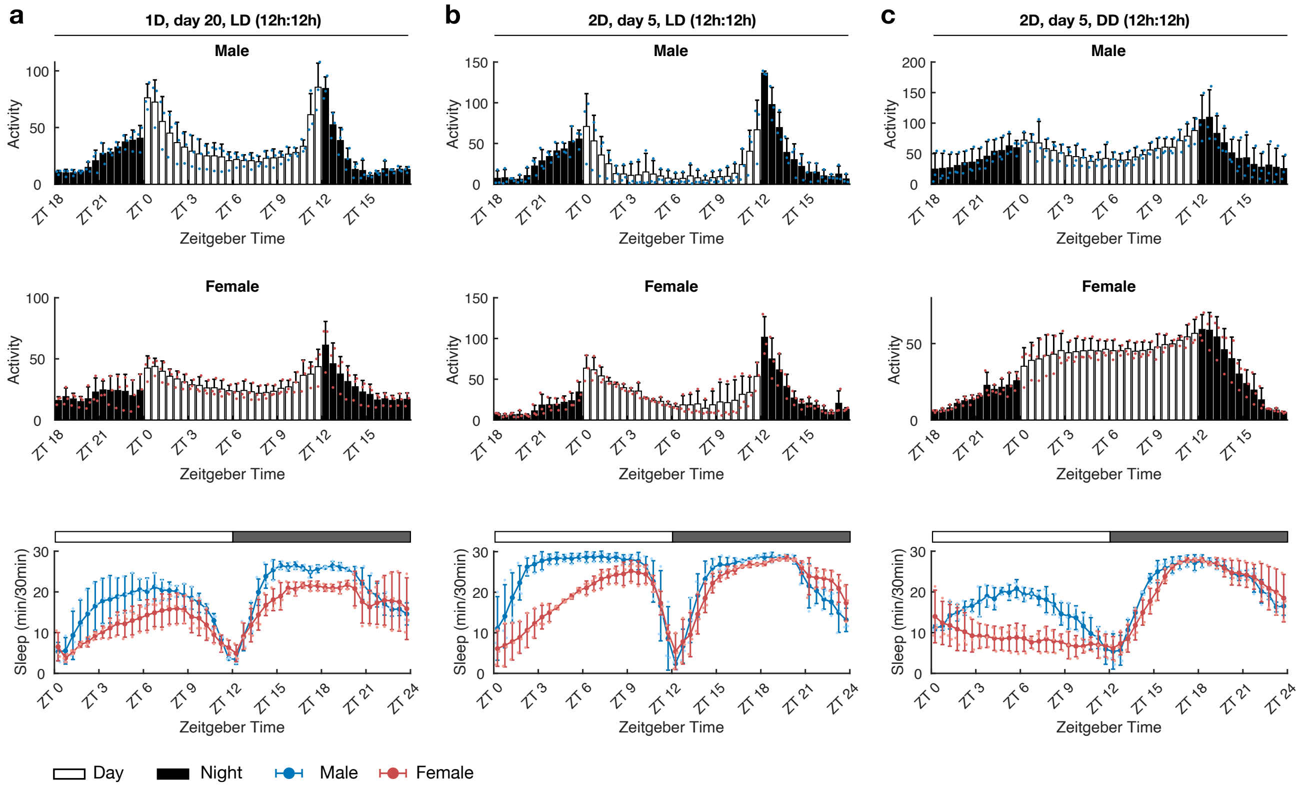

Supplementary Figure 4. Sleep pattern changes in older flies or under constant darkness.
a The statistics of circadian activities and sleep profiles of flies post eclosion 20 days in 1D chambers with LD light condition. **b** The statistics of circadian activities and sleep profiles of flies post eclosion 5 days in 2D chambers with LD light condition. **c** The statistics of circadian activities and sleep profiles of flies post eclosion 5 days in 2D chambers with constant dark condition. For **a**–**c**, the top and middle graphs show how many times the male and female flies' crossing the midline of the chambers in each time bin (30 min), respectively. The bottom graph shows how much time the flies are in a sleep bout in each 30 min. The horizontal bar indicates the light (hollow) and dark (filled) phase. Error bars indicate the SEM of biologically independent experiments.

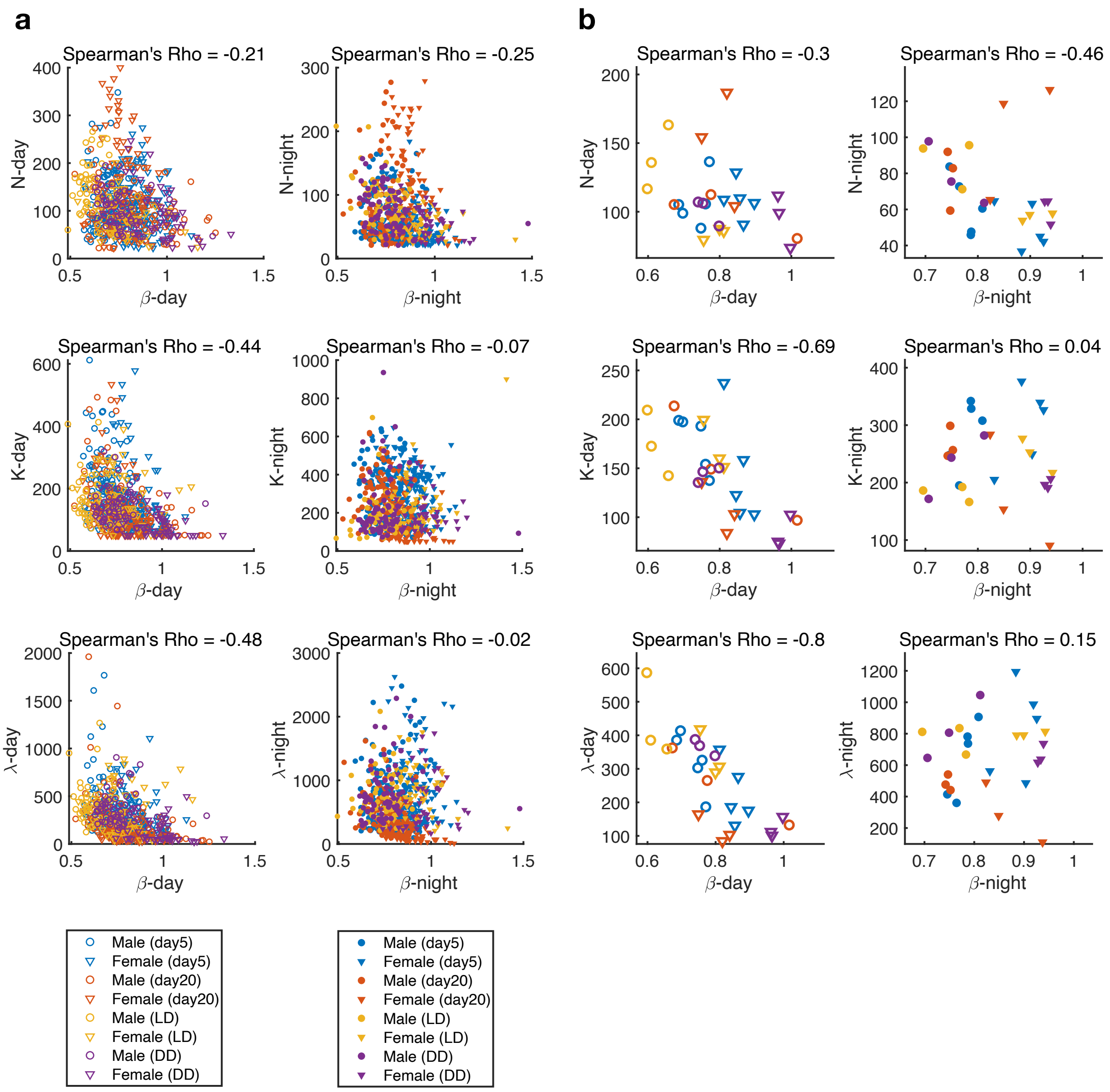

Supplementary Figure 5. Correlation between the parameters of the sleep pattern.
a–**b** Correlation of the number of sleep bout in 3 days (N) and the active index parameter (β), the sleep latency (K) and the active index parameter (β), and the sleep duration (λ) and the active index parameter (β). The Spearman's correlation coefficients are calculated with all flies' data (**a**) or with the mean of each experiment (**b**) from 4 different conditions. Each hollow marker represents one fly's parameters in the light phase (left), and each filled marker represents the data in the dark phase (right). The exact values of correlation coefficients are shown above the scatter plots.

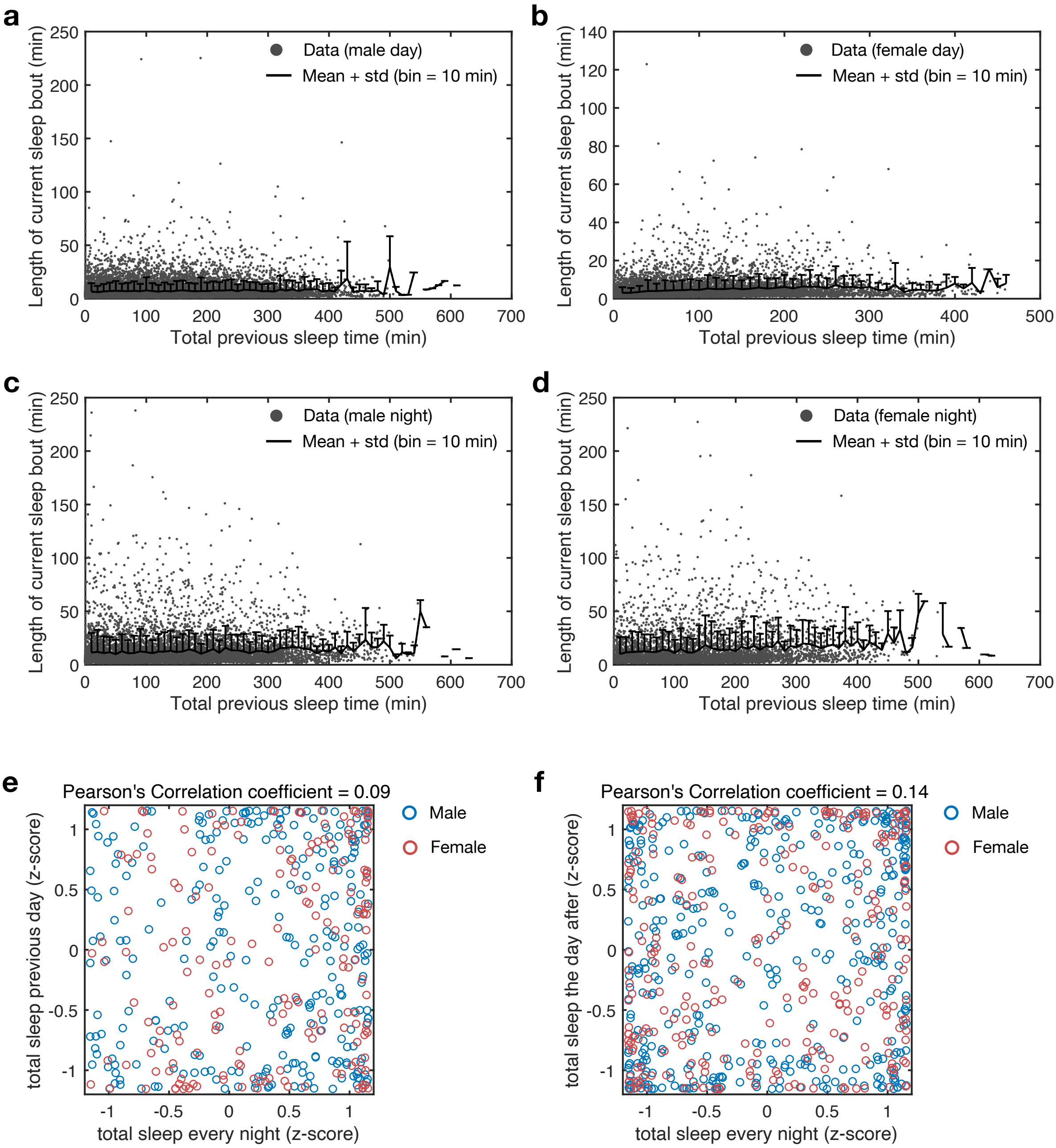

Supplementary Figure 6. Mathematical model of sleep pattern.
a–**b** The total previous sleep time does not affect the length of current sleep bout in male flies **a** or female flies **b** in the light phase. **c**–**d** The total previous sleep time does not affect the length of current sleep bout in male flies **c** or female flies **d** in the dark phase. In **a**–**d**, each grey dot represents the sum up of the sleep time before the current sleep bout, and the black curves show the mean values and standard deviations in each 10 min bin. **e**–**f** The total sleep time in the light phase and the dark phase does not show a correlation. The total sleep time is normalized with z-score for each fly in the light and dark phase, respectively. Blue circle, male; red circle, female. All the flies shown here is under the LD (12h:12h) with the 1D chambers and 5 days post eclosion.
