# Supplementary material for: Quantitative investigation reveals distinct phases in Drosophila sleep
Source: Commun Biol. 2021 Mar 19;4:364. doi: 10.1038/s42003-021-01883-y (PMC7979771; doi:10.1038/s42003-021-01883-y)
Supplement: Supplementary file 3 — Description of Additional Supplementary Files [file 42003_2021_1883_MOESM3_ESM.pdf]

Description of Additional Supplementary Files

File name: Supplementary Data 1

Description: Source Data for figures and supplementary figures. The circadian activity and sleep profile of flies, estimated parameters of sleep pattern, examples of time distribution of rest time, effects of sleep history on current sleep time, and preference of location during sleep are included in corresponding sheets.
